# BioWinfordMR: an online platform for comprehensive Mendelian randomization analysis

**DOI:** 10.3389/fimmu.2025.1695146

**Published:** 2026-01-13

**Authors:** Yunfeng Wang, Tong Wu, Xinliang Lu, Xiaoqiong Wang, Wenhua Xue

**Affiliations:** 1 The First Affiliated Hospital of Zhengzhou University, Zhengzhou, Henan, China; 2 Annoroad Gene Technology Co., Ltd., Beijing, China; 3 Department of Ultrasound, The Second Affiliated Hospital of Harbin Medical University, Harbin, Heilongjiang, China; 4 Institute of Immunology and Bone Marrow Transplantation Center of the First Affiliated Hospital, Zhejiang University School of Medicine, Hangzhou, China; 5 Department of Respiratory and Critical Care Medicine, The Second People’s Hospital of Hefei, Hefei, Anhui, China

**Keywords:** BioWinfordMR, causal inference, gut microbiota, GWAS, immune cells, Mendelian randomization, sepsis

## Abstract

**Background:**

Mendelian randomization (MR) has become a tool for the rapid and accurate identification of genetic relationships between phenotypes. However, owing to the lack of rigorous and standard analytical tools, many MR conclusions are unreproducible or controversial. Therefore, we developed the BioWinfordMR platform, which integrates curated GWAS data from multiple categories using a Shiny server to automate MR analysis.

**Method:**

We developed an online platform, BioWinfordMR, to infer the causality between sepsis, gut microbiota, and immune cells. BioWinfordMR efficiently integrates core analytical functions for MR studies. Its scientific and rigorous analytical pipeline coupled with high-performance computing capabilities from large-scale servers ensures reproducible and valid analytical conclusions. The main findings of the case study were verified using flow cytometry and real-time polymerase chain reaction (RT-PCR) analysis.

**Results:**

Through systematic analysis, we found that CD62L-CD86+ myeloid dendritic cells (DCs) were key intermediate factors that increased the risk of sepsis with enhanced gut microbiota. We also identified two risk genes, *ENTPD5* and *MANEA*, which are associated with sepsis.

**Conclusions:**

We developed a platform called BioWinfordMR to facilitate various MR analyses. BioWinfordMR ensures the accuracy and reproducibility of MR analysis and allows the discovery of potential causal relationships.

## Background

Establishing causal relationships between phenotypes represents a significant challenge in biomedical research, with profound implications for elucidating disease etiology and identifying therapeutic targets. The advent of large-scale genome-wide association studies (GWAS) has substantially advanced the field of phenome-wide causal inference over the past decade. Genetic data from millions of individuals have been sequenced, enabling a systematic analysis of associations between genetic variants and complex traits in large populations. For a given phenotype, GWAS identifies genomic loci variation associated with the trait. However, the pervasive influence of linkage disequilibrium (LD) means that the lead single-nucleotide polymorphism (SNP) within a locus often serves as a proxy for a haplotype block rather than representing the causal variant itself. Consequently, GWAS findings primarily function to delineate candidate genomic regions for subsequent investigation. This necessitates downstream fine-mapping, functional genomics interrogation—utilizing resources such as expression quantitative trait loci (eQTLs) and protein quantitative trait loci (pQTLs) —and experimental validation to ultimately pinpoint causal genes and mechanisms underlying disease, thereby informing drug target discovery. Furthermore, genetic variants identified by GWAS can be employed as instrumental variables (IVs) to infer causal relationships between phenotypes, a methodology known as Mendelian randomization (MR) (1). To facilitate this causal inference from GWAS summary statistics, a suite of statistical frameworks has been developed, capitalizing on the core principles of MR (2–5).

MR has been proposed to emulate the design of randomized controlled trials (RCTs) by utilizing genetic variants, primarily SNPs, as instrumental variables (2, 5). In this framework, genetic proxies serve as IVs to infer causal relationships between modifiable exposures and disease outcomes. Typically, a disease phenotype is conceptualized as the outcome, while a putative risk factor is treated as the exposure. The causal effect of exposure on outcome can be estimated by evaluating the association between the effect estimates of the IVs derived from two separate GWAS summary statistics.

However, accessing and processing large-scale GWAS data for MR analysis remains technically challenging, and MR methodologies often present substantial barriers for non-specialists. Many researchers, particularly those transitioning from non-quantitative disciplines into computational epidemiology, encounter significant obstacles when implementing complex MR analyses that require proficiency in programming skills and causal inference frameworks. These challenges primarily manifest in three aspects: (1) acquisition of GWAS data: Although some databases provide application programming interfaces (API) that allow users to load data online through accession identifiers, unstable network connections can lead to erroneous results, which may bias the entire research conclusion; 2) data preprocessing: Currently, the world’s largest GWAS resources include OpenGWAS (6), GWAS Catalog (7), the UK Biobank (8), and FinnGen (9). OpenGWAS and GWAS Catalog are publicly available GWAS resources. The UK Biobank and FinnGen are population-based cohorts comprising genotype data and a wide range of phenotypes. However, GWAS data from different sources do not share a consistent format. Therefore, preprocessing GWAS data from different sources poses a significant challenge for analysts; and (3) inadequate computing resources. Most GWAS data contain approximately 10 million SNP records that cannot be analyzed using conventional laptops. What is more, when conducting MR analysis on multiple GWAS, more computing resources are required. This approach is impractical for teams and individuals lacking local or cloud servers.

To address these challenges and meet the growing need for systematic curation and application of comprehensive GWAS summary data, we developed BioWinfordMR (http://biowinford.site:3838/BioWinford_MR)—an integrated platform that consolidates approximately 7,000 GWAS datasets with an intuitive web interface for interactive visualization and automated causal inference through MR methodology. The entire analytical pipeline is implemented in R, with Shiny providing the web-based frontend. Users can upload data, configure analytical parameters through an interactive interface, and submit computational tasks seamlessly. The platform supports diverse input file formats, including vcf.gz, tsv.gz, csv.gz, and various plain text formats. While VCF-formatted GWAS data is directly supported for analysis, other text-based formats require preliminary standardization through the integrated “clean format” preprocessing module. Comprehensive operational guidelines are detailed in the user guide, available for download from the BioWinfordMR website. The platform’s design incorporates all necessary components to ensure compliance with the STROBE-MR reporting guidelines.

To demonstrate the platform’s capabilities, we employed BioWinfordMR to investigate the relationship between gut microbiota and sepsis pathogenesis as a case study. Substantial evidence indicates that sepsis development is closely associated with gut microbiota (10, 11). An imbalance in the gut microbiota can induce sepsis through the destruction of the intestinal mucosal barrier function, mucosal immune function, and bacterial translocation (12). Furthermore, microbiota disturbances can trigger exaggerated inflammatory responses, leading to systemic immune dysregulation and multi-organ dysfunction (13). Using the TwoSampleMR module, we identified potential causal pathways from gut microbiota to sepsis mediated through immune cell alterations. The MR mediator module enabled the screening of statistically significant mediation pathways, while colocalization analysis and summary-data-based Mendelian randomization (SMR) were employed for potential therapeutic target identification. These integrated analytical modules collectively streamline GWAS data preprocessing and analytical procedures, facilitating novel insights that would be technically prohibitive through conventional approaches.

## Methods

### Data acquisition

We systematically collated GWAS data from prominent and emerging research domains, encompassing the gut microbiota, skin microbiota, oral microbiota, cytokine profiles, immune cell populations, metabolomic profiles, blood cell indices, mitochondrial components, and liposomal characteristics. A comprehensive summary of each curated GWAS dataset is provided in **Table 1**.

**Table 1:** Description of the collected GWAS data.

GWAS category	Number of files	Reference	Cohort
gut211	211	PMID: 33462485	18,340 individuals
gut412	412	PMID: 35115690	7,738 Dutch people
gut418	418	/	/
cytokine41	41	PMID: 33491305	8,293 Finnish individuals
cytokine91	91	PMID: 37563310	14,824 participants
Blood cell	15	PMID: 37578112	562,243 participants
Lipidome	179	PMID: 37907536	7,174 Finnish individuals
Immune cell	731	PMID: 32929287	3,757 Sardinians
Serum metabolites	1400	PMID: 36635386	8,299 Canadians
skin_Popgen	147	PMID: 36261456	273 Popgen individuals
skin_KORA	147	PMID: 36261456	324 KORA individuals
Oral microbiome	3117	PMID: 34873157	Over 1,915 individuals

Data on 211 gut microbiota were retrieved from the MiBioGen consortium with accessions GCST90016908 to GCST90017118 (14). In total, 412 gut microbiota data points were obtained from a study conducted by Esteban et al., including 207 taxa and 205 pathways representing microbial composition and function (GCST90027446–GCST90027857) (15). We also proposed a new category of gut418 consisting of 211 gut microbiota data from MiBioGen and 207 taxa data from the study by Esteban. Therefore, gut418 can be considered a more comprehensive and complete collection of the gut microbiota. GWAS data for 41 cytokines were obtained from 8,293 Finnish individuals (16). This study combined the results of the Cardiovascular Risk in Young Finns Study (YFS) and the FINRISK surveys. GWAS data for 91 plasma cytokines were obtained from a recently published study by Zhao et al. (GCST90274758–GCST90274848) (17). A total of 15 blood cell GWAS datasets were obtained from the Blood Cell Consortium meta-analysis, which included 562,243 participants (18, 19). According to the 15 blood cell GWASs, seven were related to red blood cells, six were related to white blood cells, and two were related to platelets. We collected data from 179 lipidome-related GWASs from 7,174 Finnish individuals from Ottensmann et al. (GCST90277238–GCST90277416) (20). GWAS data for 1,400 serum metabolites comprises genome-wide association studies of 1,091 blood metabolites and 309 metabolite ratios (21). A total of 731 GWASs of immune cells were retrieved from a study conducted by Valeria Orrù et al. (GCST90001391–GCST90002121) (22). GWAS data for skin microbiota were retrieved from Lucas et al. (GCST90133165–GCST90133310). Because the skin microbiota study was performed in two population-based German cohorts, we separated the data into two categories (23). Oral metagenome data from both the tongue dorsum (*n* = 2,017) and saliva (*n* = 1,915) were obtained from Liu et al. (24).

### TwoSampleMR

The foundational step in MR analysis involves estimating causal effects between phenotypic traits. Causal inference was performed using the TwoSampleMR package in R (25). Users are required to input GWAS summary statistics or accession identifiers for both the exposure and outcome variables into the TwoSampleMR module, followed by configuration of key parameters including the association *P*-value threshold, clumping distance (clump_kb), and linkage disequilibrium (LD) threshold (clump_r2). Default parameter settings were established as follows: *P*-value<5 × 10^-8^, clump_kb = 10,000, and clump_r2 = 0.001. Subsequent to parameter specification, the PLINK software was employed to perform LD-based clumping, thereby identifying independent genetic instruments (26). The F-statistic for each SNP was calculated as follows, where N and k indicate the number of participants and number of IVs, respectively. The coefficient of determination (*R*²) represents the proportion of variance in the exposure that is explained by the IVs in the regression model, ranging from 0 to 1. *R*² was calculated using the get_r_from_bsen function from the TwoSampleMR R package.


$$F\;statistic=\frac{N-k-1}{k}\times \frac{R^{2}}{1-R^{2}}$$

SNPs exhibiting an F-statistic below 10 were excluded to mitigate weak instrument bias. Following instrument selection, exposure and outcome datasets were harmonized to ensure allele orientation consistency and to remove ambiguous and palindromic SNPs. Finally, sensitivity analyses were conducted to assess heterogeneity (Cochran’s Q test), directional pleiotropy (MR-Egger intercept test), and potential reverse causation. Horizontal pleiotropy was comprehensively evaluated using the MR-PRESSO framework for outlier-corrected analysis (27). Furthermore, the availability of locally curated GWAS datasets enabled bidirectional MR analyses to investigate and account for potential reverse causal relationships.

### MVMR

The TwoSampleMR module facilitates the estimation of the direct causal effect of an exposure on an outcome. For hypothesized indirect pathways, a multivariate MR (MVMR) framework was employed to distinguish between direct effects of the exposure on the outcome and indirect effects mediated through intermediate factors (28). MVMR incorporates genetic instruments associated with multiple exposures to estimate the independent effect of each exposure on a single outcome while accounting for potential covariation among exposures (29).

The BioWinfordMR platform supports the input of multiple GWAS accession identifiers, enabling users to retrieve data via public API interfaces for integrated analysis. However, in light of occasional instability in the OpenGWAS server connectivity, we strongly recommend that users upload local summary statistics files to ensure analytic reproducibility and operational stability. Multiple exposure datasets are harmonized using the harmonize_data function to ensure allele alignment and strand consistency, followed by MVMR estimation via the mv_multiple function. Given the potential for collinearity among multiple exposures, the platform incorporates an optional feature selection procedure. When feature selection is enabled, the system automatically executes the mv_lasso_feature_selection function to identify a sparse set of relevant exposures, thereby mitigating multicollinearity and enhancing model interpretability (30).

### Mediator MR

In many scenarios, exposures influence outcomes not through direct effects, but via intermediary mediators. Two established methodologies are supported for mediation analysis within the BioWinfordMR platform. The first approach utilizes MVMR, wherein both the exposure and putative mediator are included as simultaneous predictors in a multivariate model, allowing for the estimation of their independent effects on the outcome while adjusting for mutual dependence (28). This enables direct quantification of the mediation proportion. The second methodology implements the two-step MR approach (31). This procedure first estimates the total effect of the exposure on the outcome and then sequentially assesses the effect of the exposure on the mediator and the effect of the mediator on the outcome—the product of which yields the indirect (mediated) effect. Both mediation frameworks are rigorously implemented in the BioWinfordMR platform, providing robust and flexible options for causal mediation analysis.

### Colocalization

Bayesian colocalization analysis was employed to assess the probability that two distinct phenotypes share a common causal genetic variant within a specified genomic locus (32). Colocalization was performed using the coloc R package (33). Bayesian colocalization provides the posterior probability for the five hypotheses regarding whether the two phenotypes share the same causal variant. Notably, hypothesis 3 (PPH3) reflects the posterior probability that both phenotypes are associated in the region but through distinct causal variants, whereas hypothesis 4 (PPH4) represents the posterior probability that both phenotypes are associated due to a shared causal variant. Generally, a PPH4 greater than 0.65 indicates that a potential causal mechanism is shared between the two phenotypes (34). To facilitate the interpretation of colocalization results, the LocusCompare tool was integrated to visually summarize the genetic associations and colocalization signals within the genomic region of interest.

The Coloc module supports two modes to specify the genomic locus after uploading two GWAS summary statistic files or an input of two GWAS identifiers: option 1: users could input a gene symbol, upon which the module automatically retrieves the genomic coordinates corresponding to the hg19 reference genome (35) and performs colocalization analysis within the defined gene region; option 2: users could explicitly specify a genomic region (e.g., chromosome, start, and end positions) for targeted colocalization analysis between the two traits.

### SMR

The SMR module integrates QTL data to investigate whether genetic effects on an outcome phenotype are mediated by intermediate molecular phenotypes, including gene expression, protein abundance, or DNA methylation levels (36). For gene expression mediation analysis, expression QTL (eQTL) data were obtained from the Genotype-Tissue Expression (GTEx) project, Version 8, which provides genetic instruments for gene expression across 49 distinct tissues, such as brain, heart, and whole blood (37). Methylation QTL (mQTL) data were sourced from the study by McRae et al. (38), and protein QTL (pQTL) data were acquired from the deCODE genetics repository (39).

The SMR analysis tests the association between the genetic instruments for a molecular trait (e.g., eQTLs for a gene) and the outcome trait, while the Heterogeneity in Dependent Instruments (HEIDI) test is applied to evaluate whether the observed association is likely driven by a single causal variant (pleiotropy) or multiple linked variants (linkage). A HEIDI test *P*-value greater than 0.05 indicates no significant heterogeneity, supporting the hypothesis of a shared causal variant. To account for multiple testing across numerous molecular-outcome pairs, the Benjamini–Hochberg false discovery rate (FDR) procedure was employed to adjust the raw *P*-values, thereby controlling the proportion of false-positive findings (40).

### Case study of BioWinfordMR

In this study, we present a case study of immune cell-mediated microbiota exposure that increased the risk of sepsis. This focus was selected based on substantial evidence that gut microbiota dysbiosis constitutes a significant risk factor for sepsis pathogenesis (41, 42). However, the specific mediating role of immune cells within this causal cascade remains inadequately characterized and warrants systematic validation. To address this knowledge gap, we employed the BioWinfordMR platform to rigorously delineate the mediation pathway linking gut microbiota, immune cells, and sepsis.

The GWAS summary data of sepsis were retrieved from GWAS Catalog (GCST90044692) as the outcome. This dataset comprises 1,573 European ancestry cases and 454,775 European ancestry controls. The preprocessed sepsis outcome included 11,831,932 SNPs. Genetic associations for gut microbial features were obtained from the gut418 dataset and served as exposures, while immune cell GWAS data were utilized as potential mediating variables. All GWAS data underwent a data cleaning procedure and were processed into the same input format. We performed two steps in the data cleaning process: First, we checked the data format to ensure that essential variables were present, such as SNP, effect_allele, other_allele, beta, standard error (SE), p-value, sample size, effect allele frequency (EAF), trait, and accession identifier. If the data already include SNP RSIDs, we rearrange and rename the columns to generate a standard input file. Second, if the data lacked SNP RSIDs, the BiowinfordMR platform matched the coordinates of the chromosome and position to the dbSNP database according to the corresponding reference version to obtain SNP RSIDs. During the data cleaning, we did not filter out any information. However, users can set criteria in the parameter interface for downstream analysis to filter SNPs based on parameters such as the *P*-value, LD clump, and F-statistics.

### Experimental validation

#### Mice

C57BL/6J (6–8 weeks old) were purchased from SLAC Laboratory Animal Co., Ltd. (Shanghai, China). The research protocol was reviewed and approved by the Animal Care and Use Committee of Zhejiang University School of Medicine.

#### Lipopolysaccharide-induced mouse sepsis model

The mice were randomly divided into control and sepsis groups. The control group was injected with phosphate-buffered saline (PBS), and the septic group was injected with LPS (5 mg/kg). The mice were sacrificed after 12 h, and blood, lungs, and spleen were collected for subsequent experiments.

#### Flow cytometric analysis

Single-cell suspensions prepared from the lungs or spleens were resuspended in PBS supplemented with 1% fetal bovine serum (FBS) and stained with the indicated fluorescent antibodies in the dark at 4 °C for 20 min. The cells were then washed thrice with PBS and analyzed using an ACEA NovoCyte™ system. CD62L-CD86+ DCs were gated from CD45+CD11c+ cell populations.

#### Real-time PCR analysis

Total RNA was isolated from mouse blood samples using TRIzol reagent according to the manufacturer’s instructions. Single-stranded cDNA was synthesized using cDNA Synthesis Kit. Real-time PCR was performed using the CFX Touch SYBR Green Master Mix (Bio-Rad).

#### Statistics and reproducibility

All statistical analyses were conducted using the R software (version 4.4.3). The MR analysis was performed using the TwoSampleMR R package. The significance threshold for *P*-values was set to 0.05 by default. In this study, the parameters clump_kb and clump_r2 were set to default values of 10,000 kb and 0.001, respectively. This study focused on sepsis using GWAS data from 418 gut microbiota and 731 immune cells. Gut microbiota data were derived from 18,340 individuals and 7,738 Dutch individuals. Immune cell data were obtained from 3,757 Sardinians. Sepsis data were obtained from 1,573 patients of European ancestry and 454,775 controls of European ancestry. The microbiome GWAS analysis involved participants of different genders and ages. The participants come from various countries including The Netherlands, United States, Canada, Israel, South Korea, Germany, Denmark, Belgium, Sweden, Finland, and United Kingdom. 16S ribosomal RNA (rRNA) gene sequencing profiles and genotyping data were used to uncover the associations between human genetic variants and the gut microbiome. The immune-related GWAS cohort originates from a longitudinal study ranging from 18 to 102 years, native of the central east coast of Sardinia, Italy. All participants are deeply genetically characterized, and 3,757 of them are immune-profiled.

## Results

### Flowchart of the case study

**Figure 1:**
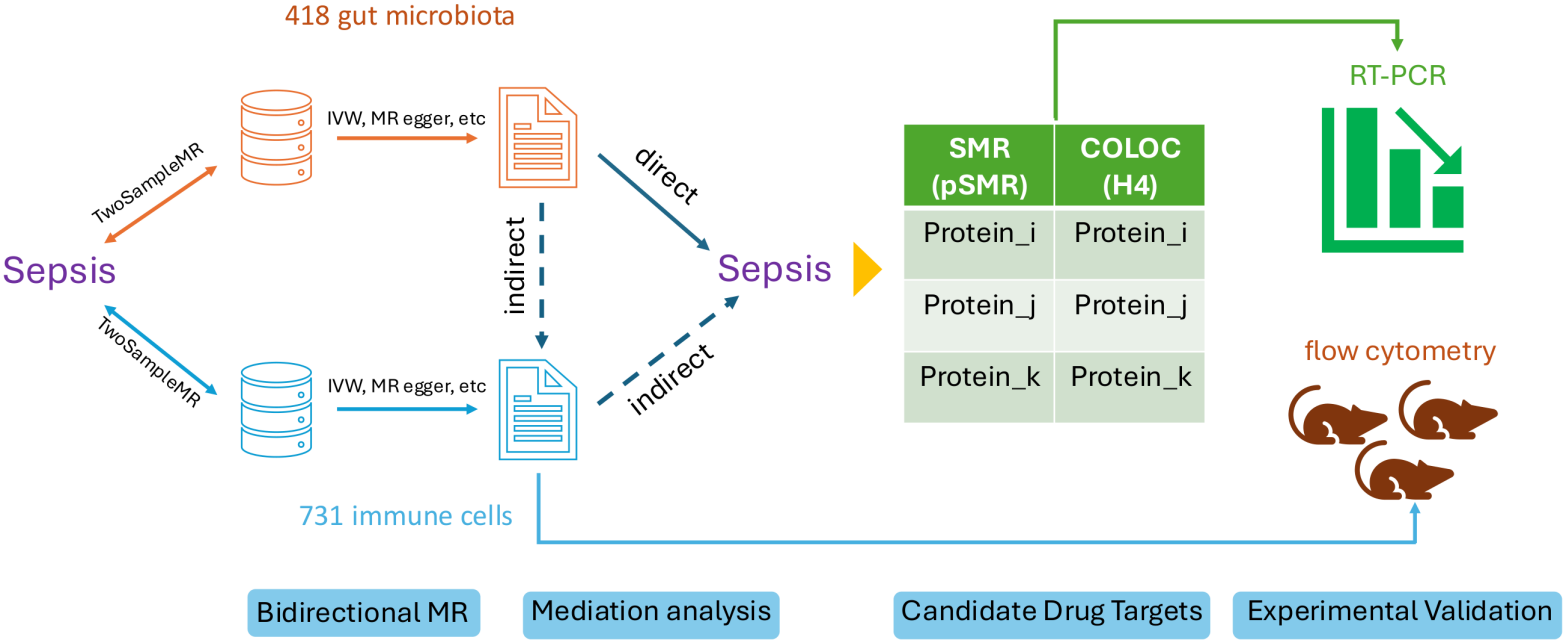
Flowchart of the case study. Significant gut microbiota and immune cell exposures were screened out using bidirectional MR analysis. The mediator immune cell was identified through a two-step mediation analysis. Candidate drug targets were predicted using SMR and colocalization. The immune cell and potential target proteins were verified using flow cytometry and RT-PCR, respectively.

The selection of an exemplar analysis was guided by two principal criteria. First, it was imperative to identify a research domain that has been extensively investigated, with well-established mechanistic underpinnings. By re-analyzing this topic using BioWinfordMR and comparing the results with those of prior studies, we could rigorously assess the scientific validity and reproducibility of the results generated by our analytical framework. Concordance with existing literature would serve to validate the methodological robustness and reliability of BioWinfordMR, while any novel findings could be further corroborated through targeted cellular experimentation. Second, we sought a research focus characterized by a sufficiently complex and multidimensional mechanistic pathway, ideally encompassing risk exposures, intermediary phenotypes (e.g., immune mediators), and clinical outcomes. This complexity was essential to fully leverage the integrated analytical capabilities of the BioWinfordMR platform, thereby demonstrating the operational efficacy and utility of its diverse modules within a biologically realistic context. Based on the two screening criteria above, we selected the immune cell-mediated impact of gut microbiota on sepsis risk as a paradigmatic research example. To enhance the clarity and accessibility of the analytical workflow for readers and potential users, we have distilled the entire process into a comprehensive flowchart (**Figure 1**). The figure outlines the following steps: (1) bidirectional MR analysis of 418 gut microbiota and sepsis associations, (2) bidirectional MR analysis of 731 immune cells and sepsis associations, (3) mediation analysis of significant microbiota–sepsis–immune cell interactions, (4) candidate drug target screening using SMR and colocalization, and (5) experimental validation of the cell and protein conclusions. We provide a detailed description of the analysis results for each step in the “Results” section.

### Gut microbiota-related exposures via bidirectional MR

In this study, we selected 418 gut microbiota-related traits from the MR Omics module as exposures. Default parameters (*P*-value<5 × 10^-8^, clump_r2 = 0.001, clump_kb = 10000, F-statistic >10) were applied to screen strong IVs. Complete details of all exposure-related SNPs are provided in **Supplementary Table S1**.

**Figure 2:**
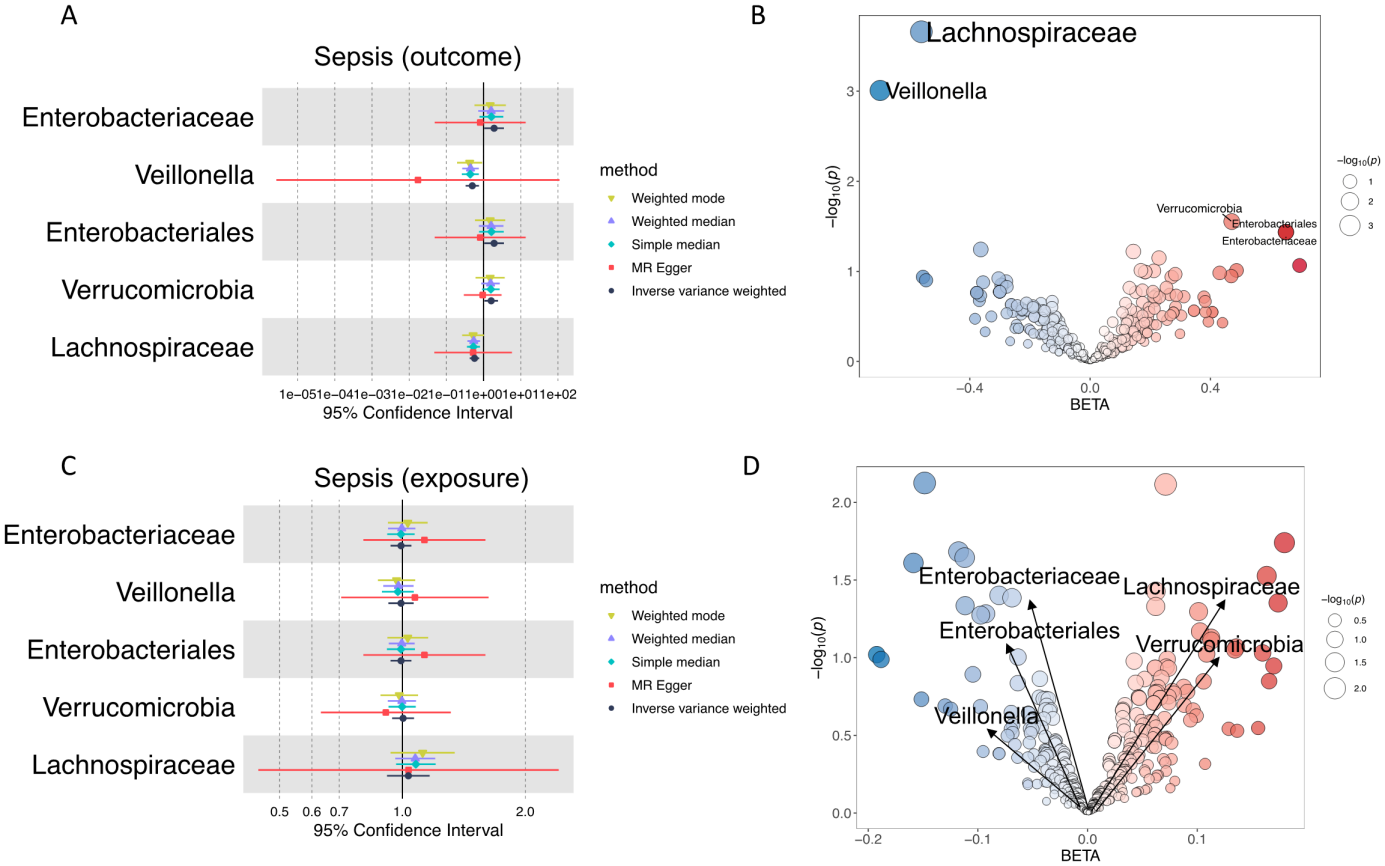
Bidirectional MR analysis between the gut microbiota and sepsis. **(A)** Forest plot of the gut microbiota as exposures. **(B)** Bubble plot of the effect of all gut microbiota as exposures. The X-axis represents the beta effect, and the Y-axis represents the -log_10_(P value). **(C)** Forest plot of gut microbiota as outcomes. **(D)** Bubble plot of the effect of all gut microbiota as outcomes.

We applied five distinct MR models for causal inference: inverse-variance weighted (MR-IVW), MR-Egger, weighted median, simple median, and weighted mode. An association was considered significant only if the effect direction was consistent across at least three models and the MR-IVW *P*-value was less than 0.05. Initial screening yielded five significant associations (**Figure 2A**). The distribution of effect estimates (beta values) and *P*-values for all microbial exposures is shown in **Figure 2B**. The results indicate negative associations between sepsis and two microbiota traits (*Veillonella* and *Lachnospiraceae*) but positive associations with *Enterobacteriaceae*, *Enterobacteriales*, and *Verrucomicrobia*.

No evidence of heterogeneity was detected among the five exposures (Cochran’s Q test *P*-value >0.05; **Supplementary Table S2**). To evaluate potential pleiotropic effects, significant associations were further scrutinized using MR-Egger and MR-PRESSO algorithms. Neither approach indicated the presence of horizontal pleiotropy (**Supplementary Tables S3**, **S4**), supporting the robustness of the causal inferences. Final MR results for the five significant gut microbiota traits with IVW *P*-value<0.05 are reported in **Table 2**. As shown in **Table 2**, *Enterobacteriaceae* and *Enterobacteriales* show identical results While the two taxa point to the same biological group in the MiBioGen consortium, they represent different taxonomic ranks (order *vs*. family). In this MR study, after IV filtering, the exposure data for both taxa corresponded to the same set of four IVs: rs111229068, rs2374342, rs62210023, and rs78143293. Consequently, the estimated effects and *P*-values calculated using these four IVs are identical. Comprehensive MR analysis results across all tested models and traits are available in **Supplementary Table S5**.

**Table 2:** MR results of the top gut microbiota with a P-value<0.05.

Exposure	Method	nsnp	OR	P-value
Enterobacteriaceae	IVW	4	1.917	0.037
Veillonella	IVW	6	0.498	0.001
Enterobacteriales	IVW	4	1.917	0.037
Verrucomicrobia	IVW	8	1.601	0.028
Lachnospiraceae	IVW	8	0.571	0

To further elucidate the causal relationship between sepsis and the gut microbiota, we conducted a bidirectional analysis with sepsis as the exposure and gut microbiota as the outcome. The results are shown in **Figure 2C** and **Table 3**.

**Table 3:** Results of reverse MR analysis using sepsis as an exposure.

Outcome	Method	nsnp	OR	P-value
Enterobacteriaceae	IVW	5	0.99	0.79
Veillonella	IVW	5	0.99	0.82
Enterobacteriales	IVW	5	0.99	0.79
Verrucomicrobia	IVW	5	1	0.91
Lachnospiraceae	IVW	4	1.03	0.59

Integrating the results of the bidirectional analysis, we did not find any gut microbiota exposures that exhibited a reciprocal causal relationship. Thus, we ultimately identified five gut microbiota exposures with causal relationships with sepsis.

### Immune-related exposures via bidirectional MR

**Figure 3:**
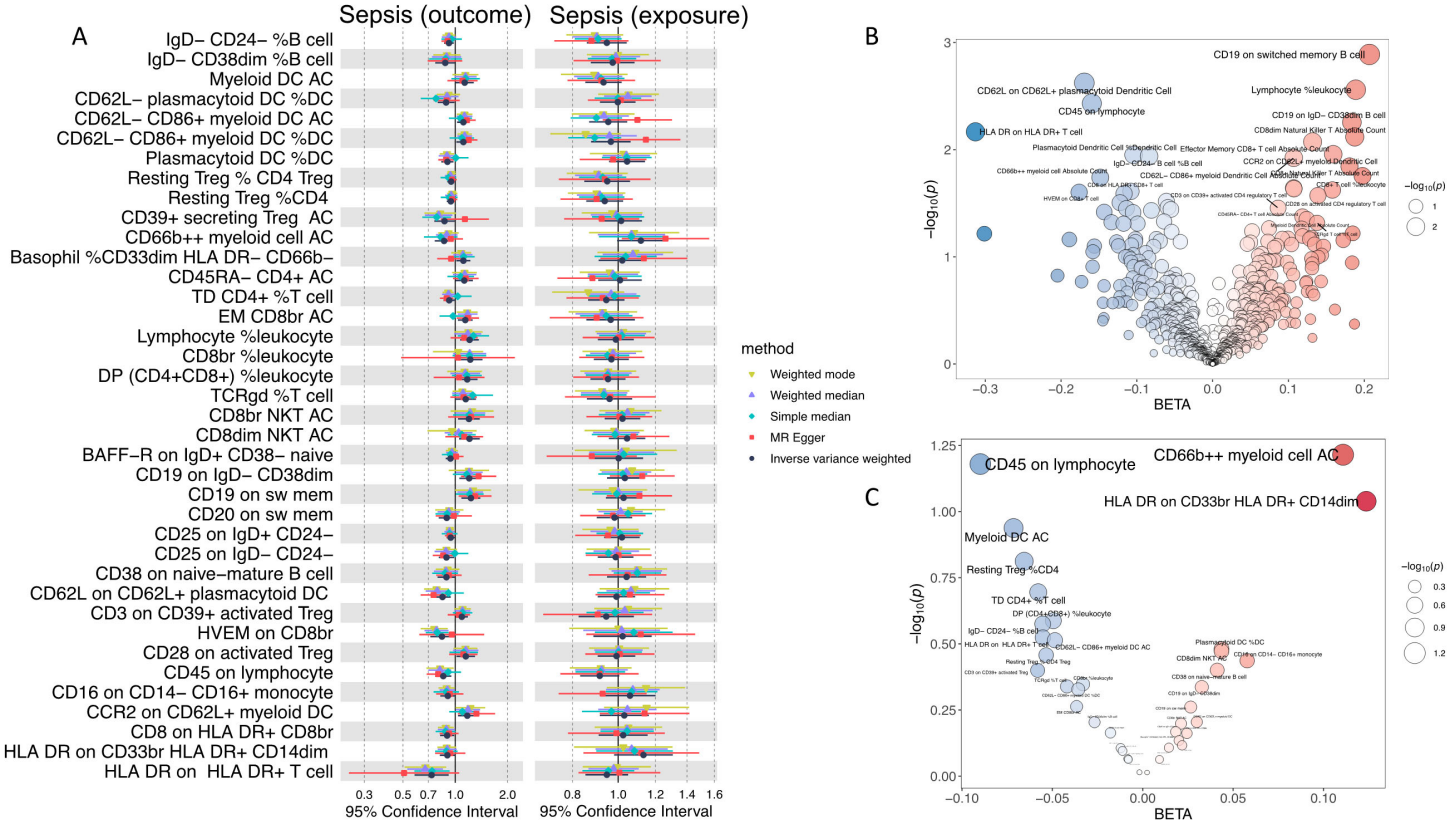
Bidirectional MR analysis between immune cell traits and sepsis. **(A)** Forest plot of immune traits as exposures (left) and outcomes (right). **(B)** Bubble plot of the effect of all immune traits as exposures. The X-axis represents the beta effect, and the Y-axis represents the -log_10_(P value). **(C)** Bubble plot of the effect of all immune traits as outcomes. The X-axis represents the beta effect, and the Y-axis represents the -log_10_(P value).

In this study, a total of 731 immune-related traits were selected from the MR Omics module as exposures, with sepsis as the outcome. The selection criteria for the IVs and analysis process were consistent with those applied in the gut microbiota analysis. Detailed information regarding the IV is available in **Supplementary Table S6**. Subsequent analysis identified 38 significant associations, visualized in a forest plot (**Figure 3A**). The distributions of beta values and *P*-values for all immune cell exposures are shown in **Figure 3B**.

Heterogeneity was assessed using Cochran’s Q test; no significant heterogeneity was detected for any of the 38 exposures (Q_p > 0.05) as documented in **Supplementary Table S7**. To evaluate potential horizontal pleiotropy, significant results were further analyzed using both MR-Egger and MR-PRESSO algorithms. Neither method provided evidence of horizontal pleiotropy (**Supplementary Tables S8**, **S9**), supporting the robustness of the causal inferences.

Associations exhibiting a *P*-value<0.01 were selected for emphasis and are presented in **Table 4**. Comprehensive MR results for all immune traits are available in **Supplementary Table S10**. Key findings indicate that elevated levels of CD19+ and CD8+ cells were significantly and positively associated with sepsis risk. Conversely, CD62L, CD45, and HLA-DR levels demonstrated significant negative correlations with sepsis.

**Table 4:** MR results of the top immune traits with P-values<0.01.

Exposure	Method	nsnp	OR	P-value
CD19 on switched memory B-cell	MR IVW	16	1.23	0.001
CD62L on CD62L+ plasmacytoid dendritic cell	MR IVW	10	0.844	0.002
Lymphocyte %leukocyte	MR IVW	12	1.208	0.003
CD45 on lymphocyte	MR IVW	12	0.853	0.004
CD19 on IgD- CD38dim B-cell	MR IVW	14	1.202	0.006
HLA DR on HLA DR+ T-cell	MR IVW	6	0.731	0.007
CD8dim natural killer T absolute count	MR IVW	18	1.206	0.008
Effector memory CD8+ T-cell absolute count	MR IVW	14	1.141	0.008

To further elucidate the directionality of the causal relationship between sepsis and immune cells, we conducted a bidirectional analysis with sepsis as the exposure and immune cells as the outcome. The results are illustrated in **Figure 3C**. The bidirectional analysis results of immune cells as outcomes are shown in **Supplementary Table S11**. According to the bidirectional MR findings, no evidence of reciprocal causality was observed between immune cell traits and sepsis. Consequently, 38 immune cell exposures were ultimately identified as exhibiting a unidirectional causal relationship with sepsis.

### Mediator analysis

**Figure 4:**
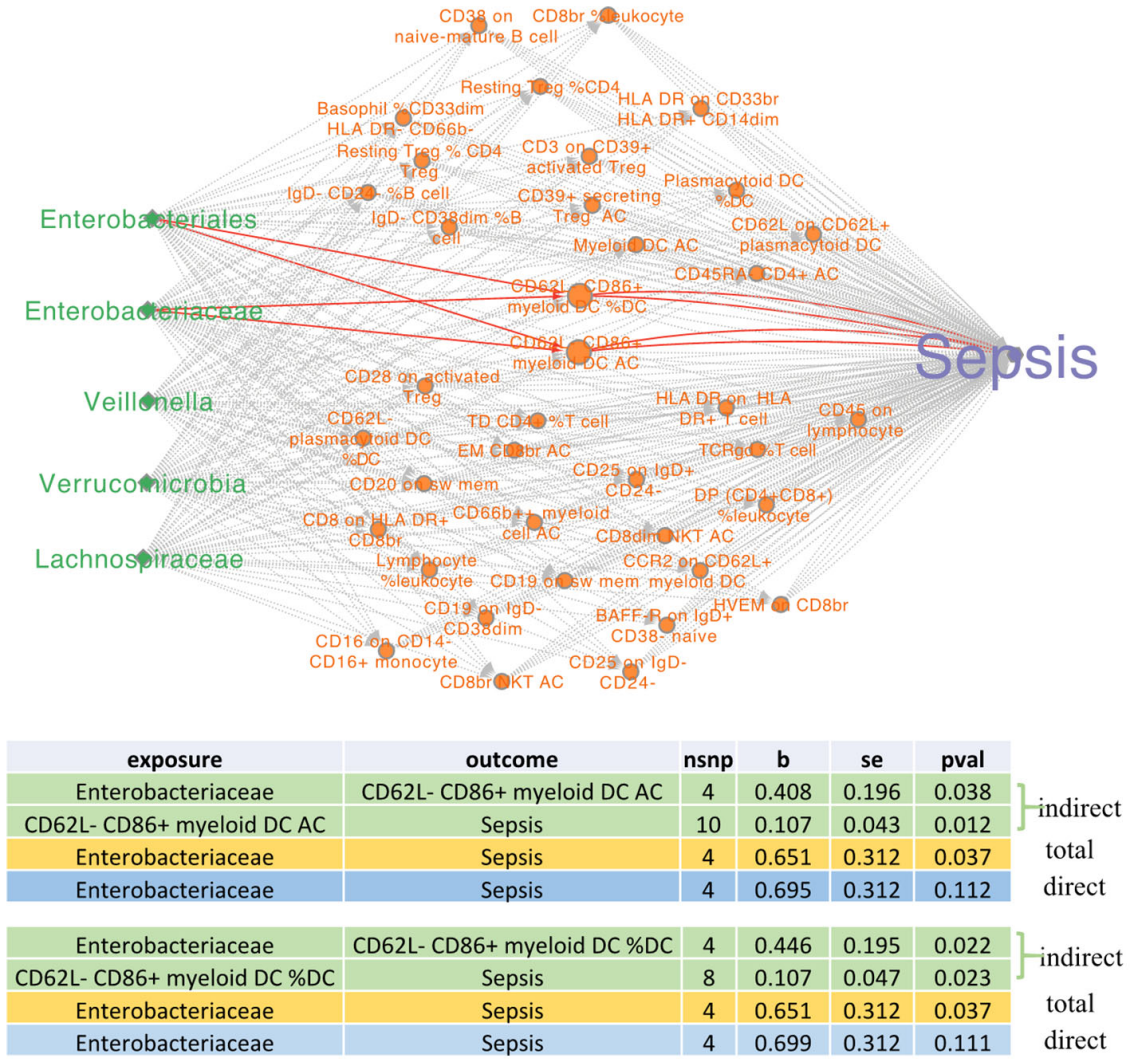
MR mediator analysis results. The green and orange nodes represent gut microbiota and immune cell GWASs related to sepsis (purple nodes). The significant mediator pathways are marked as red edges.

To further elucidate the mechanistic pathway through which gut microbiota dysbiosis influences immune regulation and exacerbates sepsis pathogenesis, we conducted the two-step mediator analysis. Within this analytical framework, gut microbiota features, immune cell traits, and sepsis status were explicitly defined as the exposure, mediator, and outcome, respectively. The Mediator_MR module integrated into the BioWinfordMR platform was utilized with default parameters (*P*-value<5 × 10^-8^, clump_kb = 10,000, clump_r2 = 0.001, F-statistic >10), and the analysis results are illustrated in **Figure 4**.

**Figure 4** delineates the results of the mediator analysis, specifying gut microbiota as the exposure, immune cells as the mediator, and sepsis as the outcome. Using a two-step MR procedure, a significant pathway was identified wherein *Enterobacteriaceae* influences sepsis risk through mediation by CD62L-CD86+ myeloid dendritic cells. The figure elaborates the indirect effects of exposure on the outcome via the mediator. It also outlines the total effect of exposure on the outcome, along with the direct effect. Both total and indirect effects were statistically significant (*P*-value<0.05). Notably, upon adjustment for the mediating effect, the direct effect became non-significant, indicating that the mediation pathway fully accounts for the causal influence—a pattern consistent with complete mediation.

### Disease-related genes via SMR

**Figure 5:**
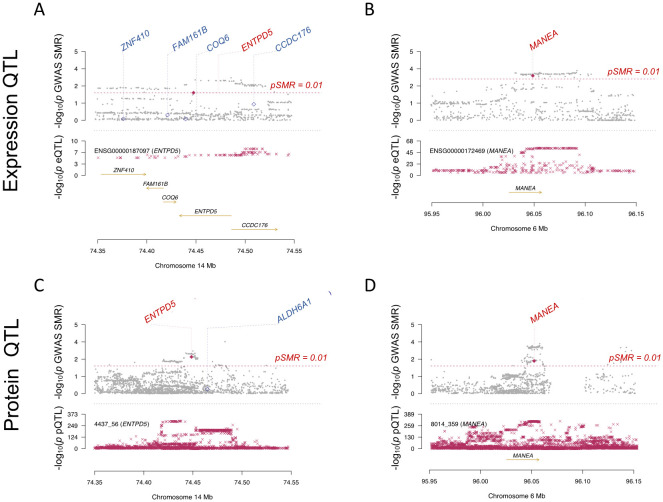
Shared significant genes between SMR.eQTL and SMR.pQTL. **(A)** SMR plot of the ENTPD5 gene using eQTLs. **(B)** SMR plot of the MANEA gene using eQTLs. **(C)** SMR plot of the ENTPD5 gene using pQTLs. **(D)** SMR plot of the MANEA gene using pQTLs.

To further elucidate the genetic determinants underlying sepsis pathogenesis, we employed the SMR module to identify genes potentially associated with sepsis susceptibility. Tissues were specified as whole blood, and eQTL data were used as exposure. A total of 40 sepsis-related genes were identified based on the criteria of pSMR<0.01 and HEIDI >0.05 (**Supplementary Table S12**). Additionally, pQTL data were analyzed, leading to the identification of 18 sepsis-related proteins (**Supplementary Table S13**). Comparative analysis of the significant findings from eQTL and pQTL analyses revealed two overlapping genes, *ENTPD5* and *MANEA*, as high-confidence candidates (**Figure 5**).

**Figures 5A, B** display the SMRplot results for *ENTPD5* and *MANEA* at the eQTL level, while **Figures 5C, D** present the SMRplot results for *ENTPD5* and *MANEA* at the pQTL level. These findings indicate that *ENTPD5* and *MANEA* are significantly associated with sepsis at both the gene and protein expression levels.

### Candidate drug target screening via colocalization

**Figure 6:**
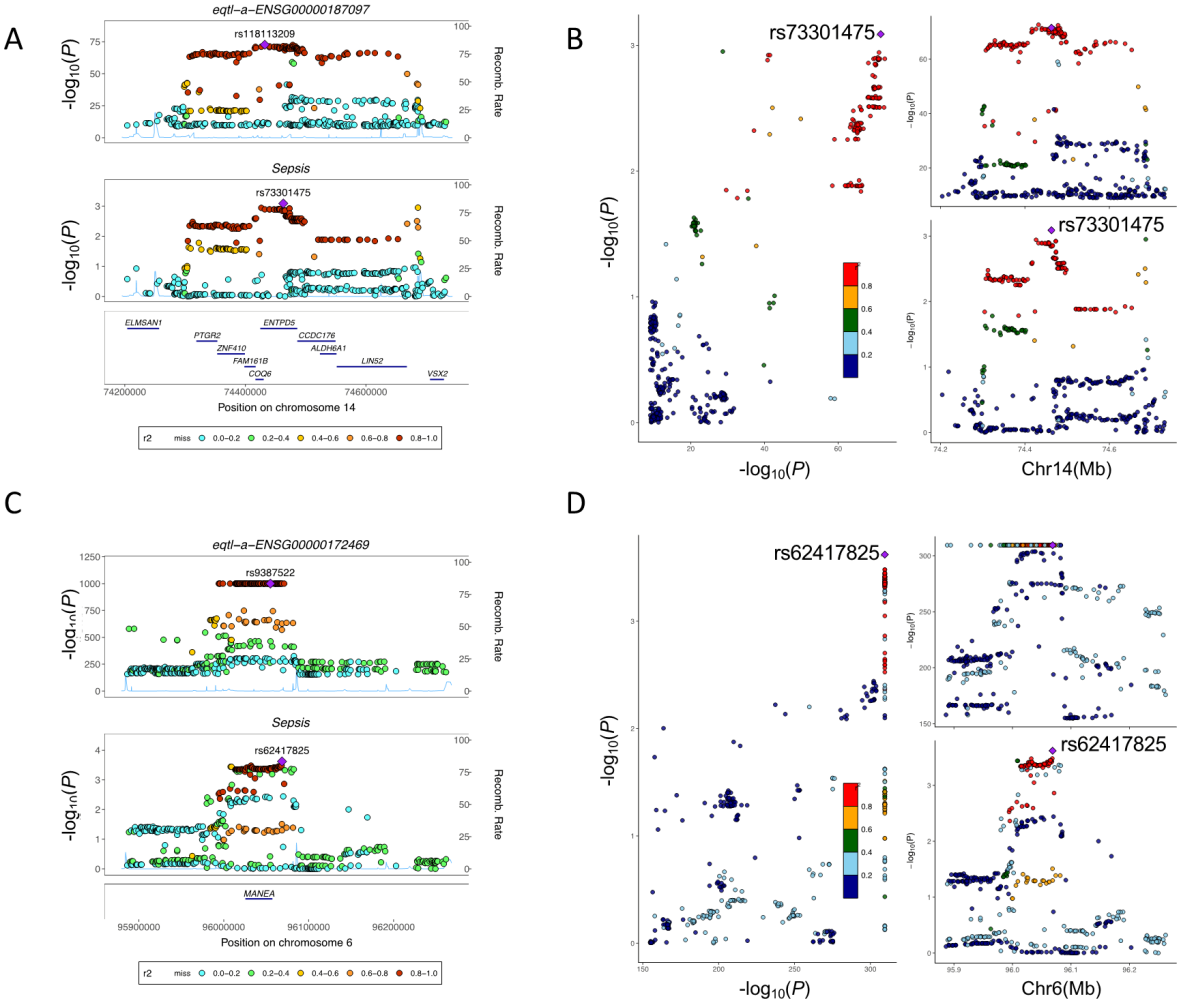
Colocalization analysis between sepsis and candidate genes. **(A)** Locus plot between sepsis and ENTPD5. The lead variant is uniquely colored purple, and all other variants are colored according to the corresponding r2 value. The recombination rate peaks are plotted in blue. All genes located in the flanking region of ENTPD5 are plotted at the bottom. **(B)** Locus comparison plots for ENTPD5. The Y-axis represents -log_10_(P values), and the X-axis represents the genome region. **(C)** Locus plot between sepsis and MANEA. **(D)** Locus comparison plots for MANEA.

To further investigate whether sepsis shares a causal variant with the candidate genes discovered by the SMR algorithm, we conducted colocalization analysis using the coloc module integrated into the BioWinfordMR platform. The eQTL GWAS summary statistics data for the two candidate genes were retrieved from OpenGWAS. The colocalization region was automatically defined based on the chromosomal coordinates of each target gene according to the GENCODE annotation database (option 1). Within each gene-specific region, colocalization analysis was conducted between the sepsis and the respective eQTL GWAS data. The results that evaluate the posterior probabilities for shared causal variation are presented in **Figure 6**.

**Figures 6A, B** present the colocalization and locus comparison results for sepsis and *ENTPD5*. **Figure 6C, D** show the colocalization and locus comparison results for sepsis and *MANEA*. The colocalization of these genes with sepsis in the PP.H4.abf group was 0.67 and 0.81, respectively. Generally, a PP.H4 value greater than 0.65 indicates a significant colocalization relationship between the two GWASs (34). Therefore, *ENTPD5* and *MANEA* share causal variants with sepsis.

### Cytometry and real-time PCR validation

**Figure 7:**
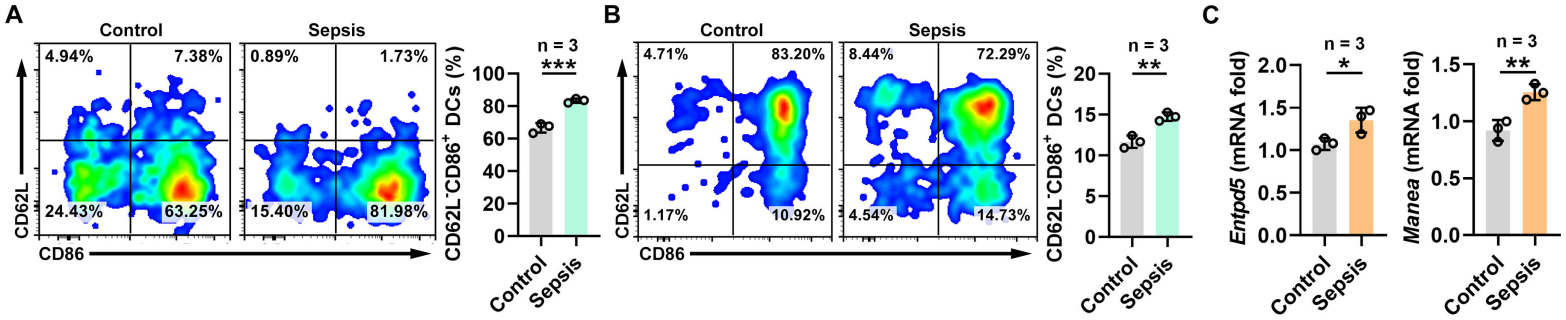
Experimental validation. **(A)** Flow cytometric analysis of CD62L-CD86+ DCs in lungs. **(B)** Flow cytometric analysis of CD62L-CD86+ DCs in spleens. **(C)** Real-time PCR analysis of Entpd5 and Manea mRNA expression in blood samples. * P<0.05, ** P<0.01, *** P<0.001.

To validate the role of CD62L-CD86+ myeloid DCs in sepsis, we used flow cytometry to validate the infiltration of CD62L-CD86+ DCs into the spleen and lung tissues of mice in both the control and sepsis groups. In the sepsis group, we observed a significantly higher proportion of CD62L-CD86+ DCs than that in the control group as shown in **Figures 7A, B**.

To validate the accuracy of the results of the BioWinfordMR platform, we experimentally verified the expression levels of the two target genes. We performed real-time PCR validation for two candidate target genes, *ENTPD5* and *MANEA*, and found a significant upregulation of these genes in the sepsis group. Real-time PCR results are included in **Figure 7C**.

## Discussion

Genetic variations not only contribute to individual phenotypic diversity but are also established as significant risk factors for a wide range of complex diseases (43). The expansion of publicly available GWAS summary datasets has facilitated the identification of numerous genetic associations between disease phenotypes and genomic variants (20, 44). MR provides a robust statistical framework to infer the causal relationships between phenotypes. However, despite offering substantial analytical opportunities, GWAS data analysis poses significant challenges for researchers in terms of methodological rigor, preprocessing complexity, and computational demands.

We developed an integrated analytical platform comprising over 7,000 preprocessed GWAS summary datasets and multiple statistical modules to facilitate systematic causal inference. This platform enables users to perform rigorous causal inference based on GWAS summary data through the following features. First, BioWinfordMR incorporates an extensive repository of preprocessed GWAS datasets that are readily accessible for analysis. The platform also includes a data preprocessing module that standardizes data from diverse sources. Second, BioWinfordMR implements state-of-the-art methodological pipelines that can be customized through user-defined parameters, thereby enhancing the reliability and reproducibility of causal inferences. Third, BioWinfordMR was deployed on a high-performance computing infrastructure, equipped with 16 cores, 64 GB of memory, and 8 TB of storage, to meet the substantial computational and storage requirements inherent to large-scale GWAS analyses.

As a practical application, we used the BioWinfordMR platform to explore potential mediation pathways through which gut microbiota influences sepsis risk via immune cell regulation. Through batch analysis using the MR Omics module, we identified five gut microbiota and 38 immune cell traits significantly associated with sepsis. The subsequent two-step analysis revealed two completely mediating pathways. Specifically, we found that *Enterobacteriaceae* positively regulate the abundance of CD62L-CD86+ myeloid DCs, which, in turn, increases sepsis risk. While prior studies have suggested potential associations between *Enterobacteriaceae*, myeloid DCs, and sepsis (45, 46), no previous research has conclusively delineated the genetic interaction pathways among these factors through MR mediator analysis. Our MR mediator analysis provides robust genetic evidence that *Enterobacteriaceae* increases sepsis risk through the activation of myeloid DCs.

Furthermore, we identified candidate genes associated with sepsis at both gene expression and protein quantitative trait levels using the SMR algorithm, followed by colocalization analysis to validate shared causal variants. Two candidate genes (*ENTPD5* and *MANEA*) were robustly associated with sepsis risk. *ENTPD5* functions as an enzyme involved in purinergic signaling and metabolism by hydrolyzing nucleoside triphosphates and diphosphates, impacting cellular processes, such as proliferation, differentiation, and survival. Mutations in the *ENTPD5* gene have been linked to certain cancers and infectious diseases (47, 48). Recent studies have shown that *ENTPD5* promotes renal injury in both humans and mouse models. Xu et al. reported that *ENTPD5* is mainly expressed in the renal tubules of the kidneys, and the expression level of *ENTPD5* is altered in mice and patients with kidney injury (49). *MANEA* encodes the enzyme mannosidase endo-alpha, which plays a crucial role in N-glycan processing in the endoplasmic reticulum. The structural characteristics of *MANEA* have inspired the development of new inhibitors that disrupt N-glycan processing and reduce pathogen infectivity in cellular models (50).

The advancement of MR methodologies has led to the development of numerous software packages tailored for MR analyses. However, these publicly available MR-related R packages primarily address discrete analytical steps rather than providing an integrated workflow. Comprehensive online platforms that consolidate extensive data resources with diverse analytical functionalities, such as BioWinfordMR, remain scarce. To objectively evaluate the capabilities of our platform, we conducted a systematic comparison with MR-Base (https://www.mrbase.org/), one of the most widely used platforms in the field (25). This comparative assessment was structured along three key dimensions: (1) accessibility and acquisition of GWAS data sources, (2) diversity and integration of analytical functions, and (3) computational efficiency and scalability.

First, regarding data acquisition, MR-Base allows users to access GWAS data online through an API, a feature shared with BioWinfordMR as both platforms are built upon the TwoSampleMR package. Although MR-Base supports the upload of user-provided summary statistics files, it imposes a strict requirement for plain-text formats containing complete information including RSID, effect_allele, beta, standard error, and *p*-values. A significant limitation arises from the fact that many GWAS datasets lack RSID information and require coordinate-based matching to a reference genome for identifier conversion. Datasets in this format cannot be processed directly on the MR-Base platform. In contrast, BioWinfordMR incorporates an automated data preprocessing module that performs this conversion seamlessly, enabling the analysis of a broader range of dataset formats.

Second, the analytical scope of MR-Base is currently confined to TwoSampleMR analysis and SNP lookup. The former is functionally and analytically consistent with the TwoSampleMR module on the BioWinfordMR platform. The latter mainly investigates linkage disequilibrium relationships near specified SNPs in order to identify proxies. BioWinfordMR, however, offers a significantly expanded suite of analytical modules not available on MR-Base, including but not limited to mediation analysis, colocalization, and multivariate MR. It is noteworthy that the developers of MR-Base explicitly acknowledge the platform’s constrained analytical scope in their documentation, stating that “This web-app represents a relatively limited analytical scope compared to using the TwoSampleMR R package.”, and recommend the direct use of the R package for advanced analyses. This presents a considerable barrier for researchers lacking computational proficiency. BioWinfordMR addresses this gap by providing an integrated, user-friendly environment that does not require programming expertise for complex analyses.

Finally, BioWinfordMR demonstrates superior computational efficiency and batch-processing capabilities. The MR-Base platform only supports single-task submissions, whether accessing data via API or uploading local files, and does not allow batch processing. In contrast, BioWinfordMR implements multithreaded parallel processing, which we have benchmarked to yield greater than fivefold increase in processing speed for equivalent tasks. Furthermore, the platform’s architecture allows users to upload a dataset once and perform batch analyses against its integrated repository of over 7,000 preprocessed GWAS datasets, significantly streamlining large-scale causal inference workflows.

The BioWinfordMR platform boasts a number of strengths as outlined below:

1. Extensive repository of preprocessed GWAS data.

Currently housing nearly 7,000 localized preprocessed GWAS datasets, BioWinfordMR enables users to access data directly via identifiers without the need to redownload from the original resource. Moreover, for non-preprocessed data from various sources, the platform offers an automated formatting module that standardizes the GWAS data into a unified format.

2. Efficient execution of MR analysis.

The sepsis case study involved over 1,000 GWAS datasets that users could download and analyze with their own laptops using the TwoSampleMR R package. However, this task is extremely labor-intensive and time-consuming. In addition to the required computational and storage resources, researchers without programming experience may introduce unknown errors during their analyses. By leveraging significant computational resources on large servers, BioWinfordMR can process data in parallel across multiple threads, thereby greatly enhancing operational efficiency and reducing processing times. Under default settings with four threads, MR analysis involving 731 immune cells was completed in approximately 17 min, whereas single-threaded analysis on a laptop took approximately 1.5 h. The platform interactively presents graphical and tabular post-analysis results, allowing users to adjust the parameters with real-time result updates within the interface.

3. Generation of reliable MR estimates.

BioWinfordMR focuses on enhancing reliability using multiple approaches to estimate pleiotropy, heterogeneity, and confounding factors. The platform offers tools, such as MR-PRESSO, to assess uncorrelated horizontal pleiotropy. Additionally, the FastTraitR (https://github.com/TullyMonster/MendelRookie) module was used to evaluate SNP confounding factors.

4. Reproducibility of MR findings.

By consolidating the data and analytical modules within a unified platform, BioWinfordMR facilitates the reproduction of results by other analysts using identical parameters. Furthermore, the platform provides R code to aid users in reproducing their results on different devices.

5. Diverse MR analysis modules.

BioWinfordMR offers comprehensive MR analysis functionalities tailored to meet user requirements for systematic and in-depth analysis. In addition to the common TwoSampleMR capabilities, the platform includes functional modules, such as MVMR (51), Mediator MR, LDSC (52), SMR, Coloc, MR Meta-Analysis, and various interactive visualization analysis modules.

Our platform, while offering substantial analytical advantages, is subject to several inherent limitations. First, GWAS summary statistics for distinct phenotypic traits may originate from either identical or partially overlapping study cohorts. Such cohort overlap can introduce systematic bias into effect estimates, potentially skewing results toward confounded observational associations (53). Although we implemented a stringent instrumental variable selection criterion to mitigate this bias, complete elimination of this bias remains challenging. Second, our platform supports multi-omics MR analysis encompassing areas such as the gut microbiota, cytokines, and immune cells. While false discovery rate (FDR) is generally recommended to control false-positives in multiple tests (54), the application of conventional FDR corrections in the MR context may be overly conservative given the anticipated sparsity of true causal relationships. Consequently, stringent multiple-testing correction might reduce power to detect true causal effects, necessitating a careful interpretation of nominally significant findings that do not survive strict FDR thresholds. Third, the statistical power of our MR analyses may be limited by the relatively small number of IVs available for some exposures, potentially affecting the robustness of the causal estimates. We minimized such effects via validation in independent datasets (eQTL and pQTL) and through experimental approaches.

Finally, while BioWinfordMR significantly enhances analytical standardization and accessibility, the appropriate interpretation of MR results remains contingent upon foundational knowledge of causal inference methodologies—particularly the core assumptions of instrumental variable analysis—and domain-specific biological expertise (e.g., sepsis immunopathology and microbial ecology). Users are strongly encouraged to consult the platform’s integrated interactive guidance modules and to contextualize findings within the broader epidemiological and mechanistic literature to ensure biologically plausible and methodologically robust conclusions.

## Data Availability

The datasets presented in this study can be found in online repositories. The names of the repository/repositories and accession number(s) can be found in the article/**Supplementary Material**.
